# Statement of Retraction: Discovery of novel benzohydroxamate-based histone deacetylase 6 (HDAC6) inhibitors with the ability to potentiate anti-PD-L1 immunotherapy in melanoma

**DOI:** 10.1080/14756366.2025.2591016

**Published:** 2025-11-29

We, the Editors and Publisher of *Journal of Enzyme Inhibition and Medicinal Chemistry*, have retracted the following article:

Peng, X., Yu, Z., Surineni, G., Deng, B., Zhang, M., Li, C., … Chen, J. (2023). Discovery of novel benzohydroxamate-based histone deacetylase 6 (HDAC6) inhibitors with the ability to potentiate anti-PD-L1 immunotherapy in melanoma. *Journal of Enzyme Inhibition and Medicinal Chemistry*, *38*(1). https://doi.org/10.1080/14756366.2023.2201408

After the publication, the authors identified errors in Figure 8A, and the following correction was published:

Correction. (2024). *Journal of Enzyme Inhibition and Medicinal Chemistry*, *39*(1). https://doi.org/10.1080/14756366.2024.2374156

Following the publication of the correction, further concerns were identified by the publisher with regards to Figure 8A. Further investigation identified concerns with Western Blot Figures 7A, 10A, and 11A. When approached for an explanation, the authors provided original data, however, concerns remain.

As the Journal and Publisher have concerns regarding the integrity of the article, we are therefore retracting the article. The authors have agreed to retract the article.

We have been informed in our decision-making by our editorial policies and the COPE guidelines.

The retracted article will remain online to maintain the scholarly record, but it will be digitally watermarked on each page as ‘Retracted’.

